# Supplementary material for: Marine n-3 Polyunsaturated Fatty Acids and Bone Mineral Density in Kidney Transplant Recipients: A Randomized, Placebo-Controlled Trial
Source: Nutrients. 2021 Jul 10;13(7):2361. doi: 10.3390/nu13072361 (PMC8308635; doi:10.3390/nu13072361)
Supplement: Supplementary file 1 [file nutrients-13-02361-s001.zip › nutrients-1272743-supplementary.pdf]

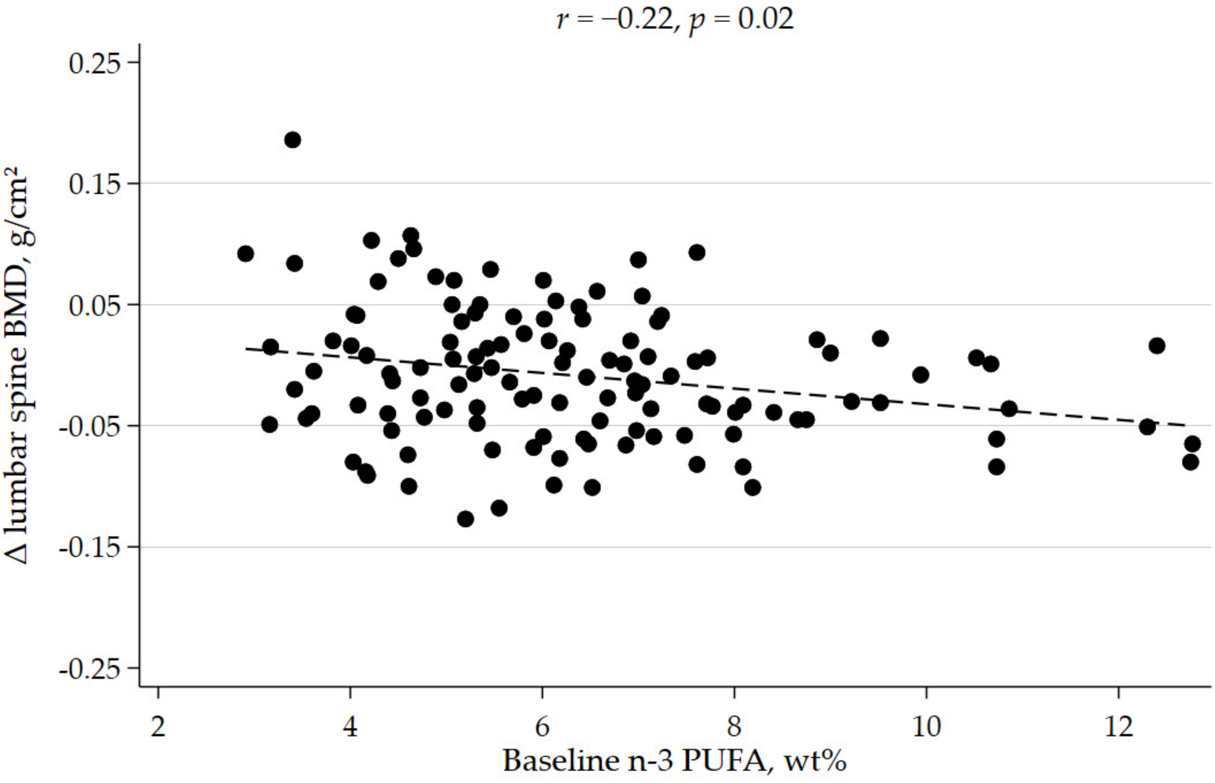

Supplementary Figure S1. The association between baseline plasma phospholipid content of n-3 PUFA and change in lumbar spine bone mineral density (BMD) during the first year post-transplant.

Supplementary Table S1. Sensitivity analysis on threshold effect of polyunsaturated fatty acids (n-3 PUFA) on bone mineral density in kidney transplant recipients.

Outcome Variable	Marine n-3 PUFA		Control		*p*
n-3 PUFA ≤6.0 wt% at baseline	*n*	Δ	*n*	Δ	
Whole body, g/cm²	29	−0.005	27	0.013	0.42
Lumbar spine, g/cm²	29	−0.019	27	0.004	0.33
Total hip, g/cm²	29	−0.001	27	0.003	0.72
Femoral neck, g/cm²	28	0.002	27	−0.008	0.45
Distal radius, g/cm²	29	0.000	27	0.023	0.11
Trabecular bone score	29	0.012	30	0.017	0.83
n-3 PUFA >6.0 wt% at follow-up					
Whole body, g/cm²	57	−0.009	32	−0.010	0.86
Lumbar spine, g/cm²	57	−0.021	32	−0.021	1.00
Total hip, g/cm²	57	0.001	33	−0.009	0.20
Femoral neck, g/cm²	56	0.000	33	−0.009	0.41
Distal radius, g/cm²	57	0.002	33	−0.007	0.47
Trabecular bone score	57	0.003	57	0.001	0.92

Data are Δ values between bone mineral density at 1 year follow-up and baseline, by intention to treat analyses, restricted to patients with below n-3 PUFA values at baseline, or above this value at follow-up.
